# Enhanced prehospital volume therapy does not lead to improved outcomes in severely injured patients with severe traumatic brain injury

**DOI:** 10.1186/s12873-019-0221-x

**Published:** 2019-01-23

**Authors:** Bjoern Hussmann, Carsten Schoeneberg, Pascal Jungbluth, Matthias Heuer, Rolf Lefering, Teresa Maek, Frank Hildebrand, Sven Lendemans, Hans-Christoph Pape

**Affiliations:** 1 Trauma Surgery Department, Alfried Krupp Hospital, Alfried-Krupp-Str. 21, 45131 Essen, Germany; 2 Department of Trauma and Hand Surgery, University Hospital, Duesseldorf, Germany; 3 Surgery Department, Philippusstift, Essen, Germany; 4 Institute for Research in Operative Medicine (IFOM), Witten/Herdecke University, Witten, Germany; 5 Clinic for Trauma and Reconstructive Surgery, University Hospital RWTH, Aachen, Germany; 6 Department of Trauma, University Hospital and University of Zurich, Zürich, Switzerland

**Keywords:** Trauma, Prehospital replacement volume, Severe traumatic brain injury, Trauma registry, Hemorrhagic shock, emergency medicine

## Abstract

**Background:**

Whether enhanced prehospital volume therapy leads to outcome improvements in severely injured patients with severe traumatic brain injury (TBI) remains controversial. The aim of this study was to investigate the influence of prehospital volume therapy on the clinical course of severely injured patients with severe TBI.

**Methods:**

Data for 122,672 patients from TraumaRegister DGU^®^ (TR-DGU) was analyzed. Inclusion criteria were defined as follows: Injury Severety Score (ISS) ≥ 16, primary admission, age ≥ 16 years, Abbreviated Injury Scale (AIS) head ≥3, administration of at least one unit of packed red blood cells (pRBCs), and available volume and blood pressure data. Stratification based on the following matched-pair criteria was performed: group 1: prehospital volumes of 0-1000 ml; group 2: prehospital volumes of ≥1501 ml; AIS head (3, 4, 5 + 6 and higher than for other body regions); age (16-54, 55-69, ≥ 70 years); gender; prehospital intubation (yes/no); emergency treatment time +/− 30 min.; rescue resources (rescue helicopter, emergency ambulance); blood pressure (20-60, 61-90, ≥ 91 mmHg); year of accident (2002-2005, 2006-2009, 2010-2012); AIS thorax, abdomen, and extremities plus pelvis.

**Results:**

A total of 169 patients per group fulfilled the inclusion criteria. Increasing volume administration was associated with reduced coagulation capability and reduced hemoglobin (Hb) levels (prothrombin ratio: group 1: 68%, group 2: 63.7%; *p* ≤ 0.04; Hb: group 1: 11.2 mg/dl, group 2: 10.2 mg/dl; *p* ≤ 0.001). It was not possible to show a significant reduction in the mortality rate with increasing volumes (group 1: 45.6, group 2: 45.6; *p* = 1).

**Conclusions:**

The data presented in this study demonstrates that prehospital volume administration of more than 1500 ml does not improve severely injured patients with severe traumatic brain injury (TBI).

## Background

For severely injured patients, the objective of prehospital volume therapy is to control bleeding and the resulting hemorrhagic shock. In such patients, uncontrollable bleeding following trauma is still considered the most common preventable cause of death [1–4]. The immediate effects of bleeding and shock may result in direct and indirect sequelae in surviving patients. For example, 20% of patients develop multi-organ failure during hospitalization, and 20% experience episodes of sepsis. Multi-organ failure and septic conditions, in addition to thromboembolic complications, increase mortality after severe trauma significantly [5]. Hence, hemorrhagic shock and its consequences represent the second most common cause of death, with severe traumatic brain injury (TBI) being the number one cause of death [6]. It is difficult to treat patients with severe TBIs in a prehospital setting. Currently, two therapy options for severe traumatic brain injuries are available: Placing the patient in a 30° semi-recumbent position and providing volume therapy.

The options for prehospital treatment of hemorrhagic shock are limited. In addition to stopping the bleeding, i.e., hemostasis of externally visible bleeding via compression (in accordance with the Advanced Trauma Life Support [ATLS^®^] guidelines), administering volume therapy is of significant importance [7]. In recent literature, the excessive non-indicated use of volume substitution in patients with severe trauma has been discussed controversially. In the late 1990s, Bickell showed that rapid transfer and modest volume therapy (accepting permissive hypotension) was useful for managing patients with penetrating trauma [8–10]. Restricted volume therapy increasingly appears to be useful for patients with blunt trauma and hemorrhagic shock [11–15]. Several publications from our group have demonstrated that extensive volume therapy is associated with an increase in mortality, even in children [16–19]. These studies showed that the coagulation status of the patients was impaired. The authors concluded that this situation may be attributed to a “dilutive effect” of excessive volume therapy.

The studies mentioned thus far almost exclusively refer to patient populations without severe TBIs. Prehospital therapy of severe TBI and hemorrhagic shock remains controversial. However, it is postulated that enhanced prehospital volume therapy should be administered in patients with severe TBI. The objective of this procedure is to minimize cerebral hypotension due to hypovolemia and subsequent cerebral hypoxia, which is associated with worse outcomes [20, 21]. However, non-indicated prehospital volume therapy leads to dilution coagulopathy [22], which in turn could also lead to worse outcomes due to the persisiting hemorrhage. A retrospective multivariate regression analysis of our working group demonstrated that the prehospital volume must be considered as independent risk factor with regard to mortality, which also applies to patients with concomitant traumatic brain injury, particularly in cases with increasing volume administration. However, this effect was less pronounced compared to patients without concomitant traumatic brain injury [23]. In recent literature, severe prehospital hemorrhage is associated with increased emergency treatment times [16]. This may lead to delayed surgery and poorer outcomes [24].

A search of the current literature raises the question whether the volume and number of substitutions in patients with severe TBI have consequences for hemorrhagic shock during the post-traumatic course. Thus, we hypothesized that enhanced prehospital volume replacement has a positive impact on the outcome of patients with severe TBI and hemorrhagic shock.

## Methods

The TraumaRegister DGU^®^ of the German Trauma Society (Deutsche Gesellschaft für Unfallchirurgie, DGU) was founded in 1993. The aim of this multi-center database was to provide anonymous and standardized documentation of severely injured patients.

The data is collected prospectively in the following four consecutive time phases from the site of the accident until discharge from the hospital: A) Prehospital phase; B) Emergency room and initial surgery; C) Intensive care unit; and D) Discharge. The documentation includes detailed information on demographics, injury pattern, co-morbidities, pre- and in-hospital management, progression in the intensive care unit, and relevant laboratory findings including data on transfusion and the outcome of each individual patient. The inclusion criterion is hospital admission via the emergency room with subsequent ICU or hospital arrival with vital signs and death before admission to the ICU. The infrastructure for documentation, data management, and data analysis is provided by the Academy for Trauma Surgery (AUC - Akademie der Unfallchirurgie GmbH), a company that is affiliated with the German Trauma Society. The scientific leadership is provided by the Committee on Emergency Medicine, Intensive Care and Trauma Management (Sektion NIS) of the German Trauma Society. The participating hospitals submit their data anonymously into a central database via a web-based application. The scientific data analysis is approved according to a peer review procedure established by Sektion NIS. The participating hospitals (90%) are primarily located in Germany; however, an increasing number of hospitals from other countries (such as Austria, Belgium, China, Finland, Luxembourg, Slovenia, Switzerland, The Netherlands, and the United Arab Emirates) also contribute data. Currently, the data for approximately 25,000 patients from more than 600 hospitals have been entered into the database annually. Participation in the TraumaRegister DGU^®^ is voluntary. For hospitals associated with the TraumaNetzwerk DGU^®^, however, the entry of at least one basic data set is obligatory for reasons of quality assurance.

The present study is consistent with the publication guidelines of the TraumaRegister DGU^®^ (TR-DGU) and is registered under the TR-DGU project ID M 2013-042.

The following patients are qualified for matching:

1. Only patients from Germany and Austria were included in this study to minimize variations related to the use of different rescue systems; all of the patients were attended by a physician before hospital admission.
2. Primary admission to the hospital (no transfers)
3. Injury Severity Score (ISS) ≥16
4. Age ≥ 16 years
5. Abbreviated Injury Scale (AIS) head ≥3
6. Infusion of at least one unit of packed red blood cells (pRBCs)
7. Data available for prehospital administered fluid volume, hemoglobin concentration on hospital admission, and blood pressure at the accident site
8. and at the time of hospital admission

**Fig. 1:**
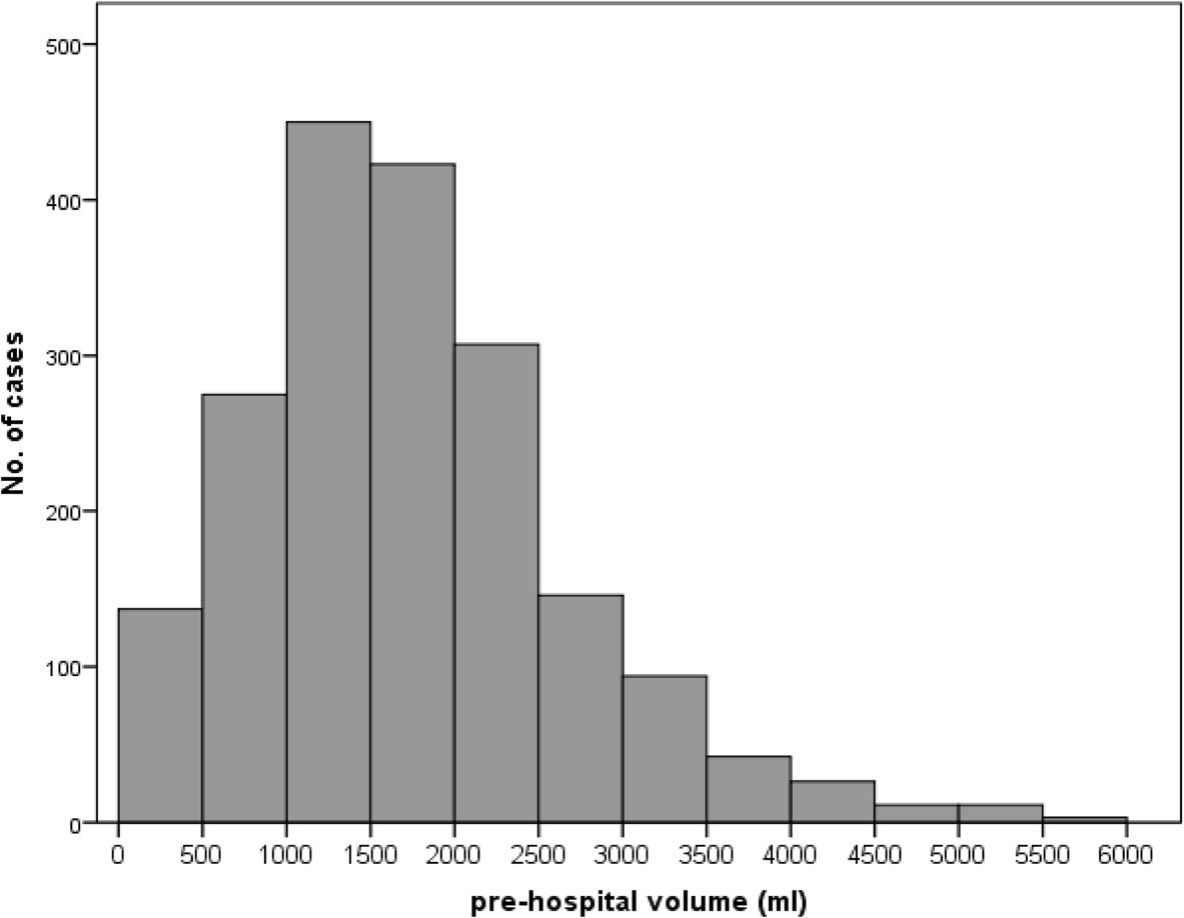
Mean value of prehospital volume of all severely injured patients with severe traumatic brain injury (TBI). 1352 patients met the inclusion criteria. Based on matching criteria, 169 of these patients in each group underwent further analysis

According to the prehospital administered fluid volume (crystalloids plus colloids), patients with severe TBI were divided into a “low-volume” (≤1000 ml) and a “high-volume” (≥1501 ml) group. This classification was chosen according to the mean value of all patients who fulfilled the inclusion criteria (Fig. 1).

To evaluate the effect of prehospital volume administration in patients with severe TBI, the patients with high- and low-volume fluid replacement were matched according to the following criteria:

- AIS head 3, 4 and 5 inclusive 6
- Pattern of injury for the following three body regions: thorax, abdomen, and extremities, including the pelvis, where the matching criteria were AIS severity ≥3 points or < 3 points; the AIS head score had to be greater than that in the other body regions
- To account for treatment changes that may have been established over the years, the date of injury was divided into three groups: (1) 2002-2005, (2) 2006-2009, (3) 2010-2012.
- Systolic blood pressure at the accident site had to be at least 20 mmHg and was subdivided into three groups with the following values: (1) 20-60 mmHg, (2) 61-90 mmHg and (3) ≥91 mmHg.
- Age categories were divided into three subgroups: (1) 16-54 years, (2) 55-69 years and (3) ≥70 years.
- Intubation (yes/no)
- Method of rescue transport (air vs. ground transport);
- Time from injury to hospital ±30 min (differences in the time from injury to hospital admission in matched patients did not exceed 10 min)
- Gender (male/female).

By applying very narrow inclusion criteria, it was intended that as many factors as possible influencing the administered volume (e.g. severity of head injury, initially measured blood pressure at the accident site, etc.) should be exactly identical in order to allow the clinical outcome to be investigated with high precision, i.e. in a statistically and clinically relevant manner. The related drastic reduction of the patient sample size was deliberately accepted.

Criteria of the definition of sepsis and single organ failure (Sequential Organ Failure Assessment Score; SOFA) were described elsewhere [25, 26]. Due to the fact, that the SOFA score is implemented by the TR-DGU^®^ involved hospitals as total value in registry, no conclusions can be drawn to the individually applied treatments and interventions. Criteria of multi-organ failure (MOF) and coagulation status (international normalized ratio; INR) were described in detail previously [17]. The Revised Injury Severity Classification (RISC) [27] was performed to assess the ISS within the groups.

### Statistics

The data were analyzed using the Statistical Package for the Social Sciences (SPSS^®^, version 17, Chicago, IL, USA). Incidences are presented as the number of cases and percentages, and continuous variables are presented as mean values with standard deviations (SD). Differences between the two matched groups were evaluated using the chi-square test in cases of categorical variables and the t-test in cases of continuous variables. In cases of obvious deviations from normality, continuous variables were tested using a non-parametric rank test (Kruskal-Wallis). A *p* value of < 0.05 was considered statistically significant.

## Results

Three hundred thirty eight patients met the inclusion criteria. There were no significant differences between any of the parameters that were used as matching criteria (e.g. AIS head; Table 1), ensuring the applicability of the statistical analysis. Furthermore, the resulting ISS – based on the AIS – did not show any significant differences (low volume, 41.4; high volume, 42.3; *p* = 0.37). This also applies to the GCS (Glasgow Coma Scale) (low volume, 6.6; high volume, 5.8; *p* = 0.11). The injury causes are presented in Table 1.

**Table 1:** Demographic and clinical data for severely injured patients with severe TBI treated before hospitalization with low- or high-volume fluid replacement therapy (169 patients per group)

Patient characteristics	Values for low- and high-volume groups			
	Low volume (0-1000 ml)	High volume (≥1501 ml)	Group mean (all patients)	*p*-values
Patients (n)	169	169	338	
Age, years				
16-54 (%)	76.9	76.9	76.9	1
55-69 (%)	8.3	8.3	8.3	1
≥ 70 (%)	14.8	14.8	14.8	1
Male (%)	82.2	82.2	82.2	0.99
Glasgow Coma Scale ≤ 8 (%)	70.1	78.0	74.1	0.10
Glasgow Coma Scale	6.6 ± 4.2	5.8 ± 3.8	6.2 ± 4.0	0.11
Injury Severity Score	41.4 ± 13.7	42.3 ± 13.6	41.8 ± 13.6	0.37
New Injury Severity Score	51.1 ± 14.8	51.9 ± 14.4	51.5 ± 14.6	0.64
Blunt trauma (%)	97.6	97.6	97.6	0.99
Cause of injury:				
Traffic accident, automobile (%)	33.1	36.8	34.9	0.16
Traffic accident, motorbike (%)	13.6	15.3	14.5	0.16
Traffic accident, bicycle (%)	6.5	9.2	7.8	0.16
Traffic accident, pedestrian (%)	15.4	16.0	15.7	0.16
Fall ≥ 3 m (%)	20.1	16.0	18.1	0.16
Fall < 3 m (%)	7.1	1.2	4.2	0.16
Other accidents (%)	4.1	5.5	4.8	0.16
Traffic accident (%)	69.8	77.9	73.8	0.10
AIS head:				
3 (%)	1.2	1.2	1.2	1
4 (%)	18.3	18.3	18.3	1
5 (%)	71.6	71.0	71.3	0.99
6 (%)	8.9	9.5	9.2	0.99
AIS thorax ≥ 2 (%)	74.0	74.0	74.0	1
AIS abdomen ≥ 2 (%)	14.8	14.8	14.8	1
AIS extremities, including pelvis ≥ 2 (%)	72.2	72.2	72.2	1

Values are the mean, standard deviation (SD) or % of the group. *AIS* Abbreviated Injury Scale, *TBI* Traumatic Brain Injury

### Prehospital and emergency department treatment

Due to the study design, significantly less prehospital volume was administered in group 1 compared to group 2 (Table 2). Similarly, a combination of crystalloid and colloidal or hyperoncotic infusions was given more often with increasing prehospital volume. Prehospital blood pressure values did not show significant differences between groups. Even on arrival in the hospital, systolic blood pressures did not show significant differences between groups (Table 2). Moreover, all other vital parameters (heart rate, respiratory rate) did not show significant differences between groups 1 and 2.

**Table 2:** Group-specific patient data for fluid administration at the accident site, in the emergency department, and during initial surgical treatment

Patient characteristics	Values for low- and high-volume groups			
	Low-volume (0-1000 ml)	High-volume (≥1501 ml)	Group mean (all patients)	*p*-values
Fluid volume replaced prehospital (ml; MV, SD)	808 ± 293.5	2098 ± 818	1453 ± 891	≤0.001
Percentage of crystalloid volume replacement solution (ml; MV, SD)	607 ± 307	1370 ± 803	991 ± 718	≤0.001
Percentage of colloidal volume replacement solution (ml; MV, SD)	186 ± 263	633 ± 477	411 ± 446	≤0.001
Percentage of hyperoncotic solutions (ml; MV, SD)	24 ± 92	95 ± 231	60 ± 180	≤0.001
Fluid volume replaced in the emergency department (ml; MV, SD)	3536 ± 2615	3116 ± 2232	3321 ± 2445	0.12
Total prehospital time (minutes; MV, SD)	66 ± 22	68 ± 21	67 ± 22	0.35
Emergency room time (minutes; MV, SD)	64 ± 36	72 ± 51	68 ± 44	0.24
BP at accident site (mmHg; MV, SD)	121 ± 30	116 ± 26	118 ± 28	0.09
BP at admission to hospital (mm Hg; MV, SD)	118 ± 34	113 ± 29	115 ± 312	0.22
BP at accident site				
20-60 mmHG (%)	2.4	2.4	2.4	1
61-90 mmHg (%)	14.8	14.8	14.8	1
≥ 91 mmHg (%)	82.8	82.8	82.8	1
Respiratory rate at the accident site (MV, SD)	13 ± 5	15	14	0.19
Heart rate at accident site (sec; MV, SD)	96 ± 25	99 ± 24	97	0.35
Heart rate at admission to hospital (sec; MV, SD)	93 ± 24	95 ± 23	94 ± 24	0.52
Hb at admission to hospital (g/dl; MV, SD)	11.2 ± 2.7	10.2 ± 2.6	10.7 ± 2.7	≤0.001
Prothrombin ratio (%) in hospital	68 ± 25.5	63.7 ± 23.3	65.8 ± 24.5	0.04
INR (MV, SD)	1.4 ± 0.9	1.5 ± 0.8	1.5 ± 0.9	0.01
Platelet count/nl at admission to hospital (MV, SD)	191,727 ± 77,362	177,982 ± 70,564	184,681 ± 74,141	0.06
Prothrombin time in hospital (sec; MV, SD)	42.8 ± 33.7	47.6 ± 32.5	45.2 ± 33.1	0.06
Base excess in hospital (mmol/l; MV, SD)	−4.9 ± 6.2	−4.2 ± 5.6	−4.5 ± 5.9	0.83
Units of pRBCs in hospital (MV, SD)	5.3 ± 5.2	6.1 ± 5.3	5.7 ± 5.3	0.07
Massive transfusions ≥ 10 units of pRBCs (%) in hospital	13.6	19.5	16.6	0.1
Units of fresh-frozen plasma in hospital (MV, SD)	3.4 ± 5.2	4.6 ± 6.1	3.9 ± 5.7	≤0.05
Prehospital use of catecholamines (%)	12.9	20.5	16.7	0.09
Prehospital chest tube (%)	4.1	9.6	6.8	0.07
Prehospital CPR (%)	5.3	5.3	5.3	1
Prehospital sedation (%)	89.1	93.8	91.5	0.21
Prehospital intubation (%)	93.5	93.5	93.5	1
Intubation in hospital (%)	94.0	95.2	94.6	0.82
Multislice CT (%)	74	72.8	73.4	0.9

Values are the mean, standard deviation (SD) or % of the group. *BP* blood pressure, *Hb* hemoglobin, *INR* International Normalized Ratio, *pRBCs* packed red blood cells, *CPR* cardiopulmonary resuscitation

All laboratory parameters (hemoglobin concentration, base excess, and coagulation values) were measured throughout the treatment in the emergency department. With the exception of prothrombin time and thrombocyte blood count, all coagulation parameters, and the Hb value were significantly reduced in group 2 (Hb: low volume, 11.2 g/dl; high volume, 10.2 g/dl; *p* ≤ 0.001, prothrombin ratio: low volume, 68.0%; high volume, 63.7%; *p* = 0.04, Table 2). There was a tendency, although not significant, of transfusing more pRBCs in the second group (low volume, 5.3 units; high volume, 6.1 units; *p* = 0.07). Also, the amount of mass transfusions was increased in group 2, but this was neither significant (low volume, 13.6%; high volume, 19.5%; *p* = 0.1). Furthermore, significantly more fresh-frozen plasma was transfused in group 2 (low volume, 3.4 units; high volume, 4.6 units; *p* ≤ 0.05, Table 2).

Patients in the second group did receive more prehospital volume therapy as well as prehospital measures, such as chest tube insertion, which was neither significant. The amount of cardiopulmonary resuscitations was not significant (Table 2).

### Clinical course and outcome

None of the groups showed significantly higher numbers of emergency surgery (including craniotomy) (low volume, 6.2%; high volume, 6.8%; *p* = 1, Table 3). There was no difference regarding the length of stay in intensive care units (ICUs) and during the entire hospitalisation, respectively. The same situation was observed with regard to the number of days of intubation in the ICU. There were no significant differences regarding the rates for sepsis, organ failure (including failure of the central nervous system), and multi-organ failure, respectively (Table 3).

**Table 3:** Clinical course and outcome of patients with severe TBI receiving low- or high-volume prehospital fluid replacement therapy after trauma

Patient characteristics	Values for low- and high-volume groups			
	Low-volume (0-1000 ml)	High-volume (≥1501 ml)	Group mean (all patients)	*p*-values
Emergency surgery (%)	6.2	6.8	6.6	1
Days in the intensive care unit (MV, SD)	16.2 ± 16.3	14.5 ± 14.4	15.3 ± 15.4	0.4
Days intubated (MV, SD)	11.9 ± 14.4	11.4 ± 12.3	11.6 ± 13.4	0.97
Organ failure (%)	77.1	83.8	80.5	0.21
Multi-organ failure (%)	61.1	67.7	64.4	0.3
Sepsis (%)	13.5	9.3	11.5	0.33
RISC prognosis (MV, SD)	44.6 ± 30	49.8 ± 29.6	47.2 ± 30.3	0.1
Died in hospital (%)	45.6	45.6	45.6	1
Died within the first 24 h (%)	23.1	24.9	24	0.8
Days of hospitalization (MV, SD)	25.1 ± 33.9	23.5 ± 31.1	24.3 ± 32.5	0.46
Glasgow Outcome Scale				
dead (%)	46.1	47.0	46.5	0.7
apallic (%)	9	7.9	8.5	0.7
strongly handicapped (%)	17.4	22.6	19.9	0.7
mildly handicapped (%)	17.4	12.8	15.1	0.7
recovered well (%)	10.2	9.8	10	0.7

Values are the mean, standard deviation (SD) or % of the group. *RISC* Revised Injury Severity Classification, *TBI* Traumatic Brain Injury

There were no significant differences regarding the mortality probability (based on the RISC prognosis) (low volume: 44.6; high volume: 49.8; *p* = 0.1, Table 3) and in terms of actual mortality (low volume: 45.6; high volume: 45.6; *p* = 1) between the two groups. Furthermore, RISC prognosis was based on values that were collected in hospital, including the prothrombin ratio, hemoglobin concentration, and administered pRBCs (27). These values have directly been influenced by the administered prehospital volume.

Moreover, the analysis of the Glasgow Outcome Scale (GOS) did not show any significant differences (Table 3).

## Discussion

Our study demonstrated no positive impact on outcome using enhanced prehospital volume therapy in bleeding patients with severe TBI. Neither mortality nor length of stay (both inhospital and ICU) differed between the two volume groups (low- and high-volume groups).

In these patients enhanced prehospital volume therapy resulted in impaired coagulation and reduced hemoglobin levels. The significantly increased administration of fresh-frozen plasma in this study also confirmed the reduced coagulation capability after enhanced prehospital volume therapy. This relationship has also been supported in studies conducted by Turner and Trunkey as well as by Geeraedts assessing blunt trauma patients without severe TBI [28–32]. The reduced coagulation capability must be rated as critical, particularly in patients after severe TBI, because bleeding is maintained and worse outcomes have been described [33].

The prevention of a second hit following primary trauma events remains the objective of prehospital therapy. As initially mentioned, the primary damage of the central nervous system cannot beimproved. Only preventive measures, such as wearing a helmet during skiing, may have a positive impact in this respect [34]. The administration of prehospital volume therapy in the most severely injured bleeding patients with severe TBI continues to be a treatment option. According to the current literature, abandoning prehospital volume therapy, as concluded by Haut et al., cannot be demanded without reservation [35]. These authors postulated that the routine use of prehospital volume replacement must be avoided because of increased mortality. As a limitation, it must be noted that the emergency system in the study by Haut differs from that in our study. Although the ISS was split into 4 groups, no organ-specific matching (e.g., using the AIS) was performed. Furthermore, the difference in mortality was only 0.3%. The maintenance of cerebral perfusion, even by means of prehospital volume administration, still represents a valid demand [36]. Regarding a complete abandonment of volume administration, valid data is not available. A prospective randomised study would be desirable, but is very difficult to conduct, due the heterogeneity of patients and due to ethical concerns. In our opinion, it would not be acceptable that one group does receive volume therapy for maintaining CPP while another group does not.

Based on our results, the maintenance of cerebral perfusion pressure can be achieved with less prehospital volume. Systemic blood pressure values were not significantly different between the two groups, indicating that the enhanced volume administration did not increase cerebral perfusion pressure but resulted in an avoidable reduction of coagulation capability. As demonstrated in previous studies of our working group and in the current literature, a restrained volume administration appears to be sufficient to achieve the blood pressure effects. A similar result was reported by Dutton et al.. These authors demonstrated that the bolus administration of volume may result in receptor-triggered blood pressure elevations [37].

Questioning the volume difference between the two groups is comprehensively discussed by our previous work [17]. In sum, a retrospective statistical analysis is not intended for investigating the physician’s individual decision at the scene. This also applies to prehospital emergency treatment time. When taking into account that delayed definitive treatment in a trauma centre may impact patient outcome significantly (golden hour of shock), the emergency treatment time of more than 60 min seems to be too high [23]. Albeit, the current average in the TraumaRegister DGU^®^ is 65 min [http://www.traumaregister-dgu.de/de/service/downloads.html]. For this reason, emergency treatment time was a matching criterion in order to establish statistical comparability.

The results of our study are supported by the current literature. In a subgroup analysis (with regard to TBI) of a prospective multi-center study, Turner et al. showed that the enhanced volume administration did not lead to positive effects [28]. Tan et al. adapted this result in their review and noted that no large-scale study exists in current literature [36]. However, they concluded that a sufficient volume must be administered to maintain cerebral perfusion pressure. Our study supports this conclusion, particularly the abandonment of volume therapy is not justified based on the current literature.

Another remarkable result of our study is that reduced volume therapy did not lead to a worse outcome in severely injured patients with severe TBI. Neither the mortality rates nor the GOS outcome (Glasgow Outcome Scale) improved in patients with higher volumes. The occurrence of multi-organ failure and organ failure, respectively, tended to differ, although this difference was not significant. Studies conducted by our working group have already demonstrated this effect in severely injured patients without severe TBI.

Interestingly, the RISC score confirmed the influence of fluid volume on mortality because this score was directly influenced by the administered prehospital volume, e.g., using the measurements of the prothrombin ratio, hemoglobin concentration or transfusion of pRCBs.

It must also be noted that some trauma patients may be transferred from other hospitals (which may not be trauma centres) as a secondary measure, i.e. after initial, potentially life-saving interventions. This population was intentionally excluded, because such interventions may influence the outcome and as such the total findings of this analysis. Moreover, the TraumaRegister DGU^®^ does not provide prehospital information on other than primarily admitted patients.

### Limitations

As we have described the underlying limitations regarding coagulation analysis, the criteria of matched-pair analysis, and the disadvantage of retrospective analysis previously [17], we would like to add here, that the TR-DGU^®^ only enrolls patients who are admitted alive to the hospital. No statements can be made with regard to patients who died at the accident site or during transport to the hospital. To a certain degree, this criterion represents a type of selection.

The systolic blood pressure measured at the accident site may also be influenced by active bleeding and, thus, cause increased prehospital volume administration. Systolic blood pressure values are usually referring to the initially measured blood pressure at the accident site, prior to interventional measures such as volume administration. In order to minimize that potential bias, we defined very narrow matching criteria in our study design. For example, total injury severity – based on ISS and NISS – has been identical. Even associated injuries have been exactly identical from a statistical point of view (e.g. AIS abdomen, thorax). Despite these strictly defined criteria, it cannot be ruled out that patients suffering from increased bleeding after initial blood pressure measurement were in one group or another. This cannot be conclusively clarified based on anonymised data in a retrospective study, but when considering the size of the population, a certain balance can be expected.

## Conclusions

The present study does not support aggressive volume replacement after trauma and bleeding in patients with severe TBI. There were no improvements of outcome or mortality due to increased prehospital volume administration. On the contrary, coagulation was worsened.

## Abbreviations

ACCP-SCCM: American College of Chest Physicians/Society of Critical Care Medicine
AIS: Abbreviated Injury Scale
ATLS: Advanced Trauma Life Support^®^
AUC: Academy for Trauma Surgery
BE: Base Excess
BP: Blood pressure
CPR: Cardiopulmonary resuscitation
DGU: German Association for Trauma Surgery
FFP: Fresh-Frozen Plasma
GCS: Glasgow Coma Scale
GOS: Glasgow Outcome Scale
Hb: Hemoglobin
ICU: Intensive Care Unit
INR: International Normalized Ratio
ISS: Injury Severity Score
MOF: Multiple organ failure
MV: Mean Value
NIS: Committee on Emergency Medicine, Intensive Care and Trauma Management
pRBC: Packed red blood cells
PTT: Prothrombin time
RISC: Revised Injury Severity Classification
RR: Respiratory rate
SD: Standard Deviation
SOFA: Sequential Organ Failure Assessment
SPSS^®^: Statistical Package for the Social Sciences
TBI: Traumatic Brain Injury

## References

[1] Hess JR, Brohi K, Dutton RP, Hauser CJ, Holcomb JB, Kluger Y (2008). The coagulopathy of trauma: a review of mechanisms. J Trauma.

[2] Teixeira PG, Inaba K, Hadjizacharia P, Brown C, Salim A, Rhee P, Browder T, Noguchi TT, Demetriades D (2007). Preventable or potentially preventable mortality at a mature trauma center. J Trauma.

[3] Gruen RL, Jurkovich GJ, McIntyre LK, Foy HM, Maier RV (2006). Patterns of errors contributing to trauma mortality: lessons learned from 2,594 deaths. Ann Surg.

[4] Søreide K, Krüger AJ, Vårdal AL, Ellingsen CL, Søreide E, Lossius HM (2007). Epidemiology and contemporary patterns of trauma deaths: changing place, similar pace, older face. World J Surg.

[5] Lendemans S, Kreuzfelder E, Waydhas C, Nast-Kolb D, Flohé S (2004). Clinical course and prognostic significance of immunological and functional parameters after severe trauma. Unfallchirurg.

[6] Sauaia A, Moore FA, Moore EE, Moser KS, Brennan R, Read RA (1995). Epidemiology of trauma deaths: a reassessment. J Trauma.

[7] American College of Surgeons Committee on Trauma (2012). ATLS student course manual.

[8] Bickell WH, Stern S (1998). Fluid replacement for hypotensive injury victims: how, when and what risks?. Curr Opin Anaesthesiol.

[9] Bickell WH, Barrett SM, Romine-Jenkins M, Hull SS, Kinasewitz GT (1994). Resuscitation of canine hemorrhagic hypotension with large-volume isotonic crystalloid: impact on lung water, venous admixture, and systemic arterial oxygen saturation. Am J Emerg Med.

[10] Bickell WH (1993). Are victims of injury sometimes victimized by attempts at fluid resuscitation?. Ann Emerg Med.

[11] Curry N, Davis PW (2012). What’s new in resuscitation strategies for the patient with multiple trauma?. Injury.

[12] Geeraedts LM, Pothof LA, Caldwell E, de Lange-de Klerk ES, D'Amours SK (2015). Prehospital fluid resuscitation in hypotensive trauma patients: do we need a tailored approach?. Injury.

[13] Albreiki M, Voegeli D (2018). Permissive hypotensive resuscitation in adult patients with traumatic haemorrhagic shock: a systematic review. Eur J Trauma Emerg Surg.

[14] Dula DJ, Wood GC, Rejmer AR, Starr M, Leicht M (2002). Use of prehospital fluids in hypotensive blunt trauma patients. Prehosp Emerg Care.

[15] Talving P, Palstedt J, Riddez L (2005). Prehospital management and fluid resuscitation in hypotensive trauma patients admitted to Karolinska University hospital in Stockholm. Prehosp Disaster Med.

[16] Hussmann B, Taeger G, Lefering R, Waydhas C, Nast-Kolb D, Ruchholtz S, et al.; TraumaRegister der Deutschen Gesellschaft für Unfallchirurgie. Lethality and outcome in multiple injured patients after severe abdominal and pelvic trauma: influence of preclinical volume replacement - an analysis of 604 patients from the trauma registry of the DGU. Unfallchirurg 2011;114(8):705-712. 10.1007/s00113-010-1842-4 21152886

[17] Hussmann B, Lefering R, Waydhas C, Touma A, Kauther MD, Ruchholtz S, et al.; Trauma Registry of the German Society for Trauma Surgery. Does increased prehospital replacement volume lead to a poor clinical course and an increased mortality? A matched-pair analysis of 1896 patients of the trauma registry of the German Society for Trauma Surgery who were managed by an emergency doctor at the accident site. Injury 2013;44(5):611-617. 10.1016/j.injury.2012.02.004 22377276

[18] Huβmann B, Lefering R, Taeger G, Waydhas C, Ruchholtz S, Sven Lendemans and the DGU Trauma Registry (2011). Influence of prehospital fluid resuscitation on patients with multiple injuries in hemorrhagic shock in patients from the DGU trauma registry. J Emerg Trauma Shock.

[19] Hussmann B, Lefering R, Kauther MD, Ruchholtz S, Moldzio P, Lendemans S, TraumaRegister DGU® (2012). Influence of prehospital volume replacement on outcome in polytraumatized children. Crit Care.

[20] Jagoda A, John Bruns J, Leon-Carrion J, Wild KRH, Zitney GA (2006). Prehospital management of traumatic brain injury. Brain injury treatment: theories and practices.

[21] Prabhakar H, Sandhu K, Bhagat H, Durga P, Chawla R (2014). Current concepts of optimal cerebral perfusion pressure in traumatic brain injury. J Anaesthesiol Clin Pharmacol.

[22] Christensen EF, Deakin CD, Vilke GM, Lippert FK, Wilson WC, Grande CM, Hoyt DB (2007). Prehospital care and trauma systems. Trauma: emergency resuscitation perioperative Anaesthesia surgical management.

[23] Hussmann B, Heuer M, Lefering R, Touma A, Schoeneberg C, Keitel J, Lendemans S (2015). Prehospital volume therapy as an independent risk factor after trauma. Biomed Res Int.

[24] Hartings JA, Vidgeon S, Strong AJ, Zacko C, Vagal A, Andaluz N, et al., Co-Operative Studies on Brain Injury Depolarizations. Surgical management of traumatic brain injury: a comparative-effectiveness study of 2 centers. J Neurosurg 2014;120(2):434-446. 10.3171/2013.9.JNS13581 24180566

[25] Levy MM, Fink MP, Marshall JC, Abraham E, Angus D, Cook D (2003). 2001 SCCM/ESICM/ACCP/ATS/SIS International Sepsis Definitions Conference. Crit Care Med.

[26] Vincent JL, Moreno R, Takala J, Willatts S, De Mendonca A, Bruining H (1996). The SOFA (Sepsis-related organ failure assessment) score to describe organ dysfunction/failure. On behalf of the working group on Sepsis-related problems of the European Society of Intensive Care Medicine. Intensive Care Med.

[27] Lefering R (2009). Development and validation of the revised injury severity classification (RISC) score for severely injured patients. Europ J Trauma Emerg Surg.

[28] Turner J, Nicholl J, Webber L, Cox H, Dixon S, Yates D (2000). A randomized controlled trial of prehospital intravenous fluid replacement therapy in serious trauma. Health Technol Assess.

[29] Trunkey DD (2001). Prehospital fluid resuscitation of the trauma patient. An analysis and review. Emerg Med Serv.

[30] Geeraedts LM, Kaasjager HA, van Vugt AB, Frölke JP (2009). Exsanguination in trauma: a review of diagnostics and treatment options. Injury.

[31] Soudry E, Stein M (2004). Prehospital management of uncontrolled bleeding in trauma patients: nearing the light at the end of the tunnel. Isr Med Assoc J.

[32] Pepe PE, Mosesso VN, Falk JL (2002). Prehospital fluid resuscitation of the patient with major trauma. Prehosp Emerg Care.

[33] de Oliveira Manoel AL, Neto AC, Veigas PV, Rizoli S (2015). Traumatic brain injury associated coagulopathy. Neurocrit Care.

[34] Sulheim S, Holme I, Ekeland A, Bahr R (2006). Helmet use and risk of head injuries in alpine skiers and snowboarders. JAMA.

[35] Haut ER, Kalish BT, Cotton BA, Efron DT, Haider AH, Stevens KA (2011). Prehospital intravenous fluid administration is associated with higher mortality in trauma patients: a National Trauma Data Bank analysis. Ann Surg.

[36] Tan PG, Cincotta M, Clavisi O, Bragge P, Wasiak J, Pattuwage L (2011). Review article: prehospital fluid management in traumatic brain injury. Emerg Med Australas.

[37] Dutton RP, Mackenzie CF, Scalea TM (2002). Hypotensive resuscitation during active hemorrhage: impact on in-hospital mortality. J Trauma.

